# Upregulation of miR-133b and miR-328 in Patients With Atrial Dilatation: Implications for Stretch-Induced Atrial Fibrillation

**DOI:** 10.3389/fphys.2019.01133

**Published:** 2019-09-10

**Authors:** Michela Masè, Margherita Grasso, Laura Avogaro, Manuel Nicolussi Giacomaz, Elvira D’Amato, Francesco Tessarolo, Angelo Graffigna, Michela Alessandra Denti, Flavia Ravelli

**Affiliations:** ^1^Laboratory of Biophysics and Biosignals, University of Trento, Trento, Italy; ^2^Healthcare Research and Innovation Program, Bruno Kessler Foundation, Trento, Italy; ^3^Department of Cellular, Computational and Integrative Biology, University of Trento, Trento, Italy; ^4^Division of Cardiac Surgery, Santa Chiara Hospital, Trento, Italy

**Keywords:** chronic stretch, microRNA, atrial fibrillation, arrhythmic substrate, post-transcriptional regulation, atrial remodeling

## Abstract

Atrial stretch and dilatation are common features of many clinical conditions predisposing to atrial fibrillation (AF). MicroRNAs (miRs) are emerging as potential molecular determinants of AF, but their relationship with atrial dilatation (AD) is poorly understood. The present study was designed to assess the specific miR expression profiles associated with AD in human atrial tissue. The expressions of a preselected panel of miRs, previously described as playing a role in cardiac disease, were quantified by reverse transcription-quantitative polymerase chain reaction (RT-qPCR) in atrial tissue samples from 30 cardiac surgery patients, who were characterized by different grades of AD and arrhythmic profiles. Our results showed that AD *per se* was associated with significant up-regulation of miR-328-3p and miR-133b (*p* < 0.05) with respect to controls, with a fold-change of 1.53 and 1.74, respectively. In a multivariate model including AD and AF as independent variables, miR-328-3p expression was mainly associated with AD grade (*p* < 0.05), while miR-133b was related to both AD (*p* < 0.005) and AF (*p* < 0.05), the two factors exerting opposite modulation effects. The presence of AF was associated with significant (*p* < 0.05) up-regulation of the expression level of miR-1-3p, miR-21-5p, miR-29a-3p, miR-208b-3p, and miR-590-5p. These results showed the existence of specific alterations of miR expression associated with AD, which may pave the way to future experimental studies to test the involvement of post-transcriptional mechanisms in the stretch-induced formation of a pro-arrhythmic substrate.

## Introduction

Atrial fibrillation (AF) is the most frequent sustained cardiac arrhythmia seen in the clinical practice, and is associated with pronounced cardiovascular morbidity and mortality, mostly due to an increased risk of stroke (Chugh et al., 2014). AF is a multi-factorial disease, which often occurs in presence of underlying cardiac abnormalities and is supported by changes in electrophysiological, anatomical, and structural properties, generally referred to as atrial remodeling (Schotten et al., 2011). Abnormal substrates are commonly encountered in different conditions that predispose to AF, such as hypertension and heart failure, whereby atrial stretch plays a key mechanistic role (Ravelli and Allessie, 1997; Allessie et al., 2002; Kohl and Ravens, 2003; Ravelli, 2003; Schotten et al., 2003). On the other hand, AF persistence itself can lead to atrial dilatation (AD) and atrial wall stretch (Allessie et al., 2002).

Compelling clinical and experimental evidence demonstrate the contribution of atrial stretch to the different AF mechanisms (Ravelli, 2003; Schotten et al., 2003). Atrial stretch may induce afterdepolarizations promoting focal activity, and may increase the atrial surface, shorten the refractory period and impair conduction, thus favoring reentrant arrhythmias (Ravelli, 2003). Chronic AD may further contribute to the formation of an electroanatomic substrate by activation of numerous signaling pathways leading to cellular hypertrophy, fibroblast proliferation, and tissue fibrosis (Schotten et al., 2003).

Despite the evidence supporting the role of stretch in AF, the precise pathophysiological mechanisms underlying the phenomenon, and in particular the cellular pathways and molecular determinants of the macroscopic changes induced by stretch, remain to be fully elucidated (Van Wagoner, 2008). A growing body of work has recently suggested the role of microRNAs (miRs), small non-coding RNAs, as potential molecular mediators in the electrophysiological and structural remodeling maintaining AF (Santulli et al., 2014; Luo et al., 2015; van den Berg et al., 2017). Additionally, exosomal miRs are emerging as key mediators of cell-to-cell communication (Santulli, 2018), playing a role in myocardial injury, repair, and regeneration (Zhang et al., 2017).

Despite the growing evidence on the role of miRs in cardiac disease, up to now no study has investigated the potential involvement of miRs in atrial stretch – AF interactions. In order to address this issue, the present study was designed to characterize the specific miR expression profiles associated with human AD. To this aim the expression of a panel of twelve miRs (Supplementary Table S1), previously indicated as having a role in cardiovascular disease and AF remodeling (Santulli et al., 2014; Luo et al., 2015; van den Berg et al., 2017), was quantified by reverse transcription-quantitative polymerase chain reaction (RT-qPCR) in human atrial tissue samples from cardiac surgery patients, characterized by different grades of AD and arrhythmic profiles.

### Study Population and Design

Tissue samples were excised from the right atrial appendage in 30 patients (six females, median age of 73.5 years, interquartile range (IQR): 68–77 years) undergoing aortic or mitral valve replacement with extracorporeal circulation at Santa Chiara Hospital in Trento. Nine patients had documented persistent AF (five long-standing persistent AF, AF ≥ 1 year), while the remaining patients presented no history of AF. The median AF duration was 36 months (range 2–156 months). In all patients basic demographic and clinical information, including sex, age, valvular disease and associated comorbidities was acquired (Table 1). All nine patients with mitral valve disease suffered from mitral regurgitation and four had also stenosis. Most patients were receiving pharmacological agents, including cardiovascular drugs (beta-blockers, calcium antagonists, ACE inhibitors, and digitalis), which were, whenever possible, withdrawn before surgery. Preoperative 2-dimensional transthoracic echocardiography was performed routinely in all patients. In each patient atrial size (atrial linear dimensions, area and/or atrial volume) was measured (Lang et al., 2015), and the level of left atrial enlargement was graded according to the criteria reported in Supplementary Table S2 (Lang et al., 2006), i.e., grade 0, reference range/normal; grade 1, mildly abnormal; grade 2, moderately abnormal; grade 3, severely abnormal. Atrial echocardiographic dimensions and AD grades for each patient are reported in Supplementary Table S3. It is worth noticing that in the subgroup of patients where right atrial dimensions were available right atrial enlargement significantly correlated with the left one (*r* = 0.95, *p* < 0.0001) (Supplementary Figure S1).

**TABLE 1:** Demographic and clinical description of the patient population.

	**Ctrl group (*n* = 12)**	**AD group (*n* = 9)**	**AF group (*n* = 9)**	***p*-values**
Age (years)	74 [64–76.5]	72 [65.5–81.2]	74 [70.2–76.2]	NS (0.90)
Men, n (%)	10 (83.3)	8 (88.9)	6 (66.7)	NS (0.47)
Female, n (%)	2 (16.7)	1 (11.1)	3 (33.3)	NS (0.47)
LA enlargement grade	**1 [0**–**1]**	**3 [2**–**3]**	**3 [3**–**3]**	**< 0.0001**
LVEF,%	63.8 ± 4.9	59.4 ± 10.3	56.6 ± 13.3	NS (0.24)
AF duration (months)	–	–	36 [4.3–109.5]	–
Comorbidities				
Aortic valve disease, n (%)	10 (83.3)	6 (66.7)	6 (66.7)	NS (0.60)
Mitral valve disease, n (%)	2 (16.7)	2 (22.2)	5 (55.6)	NS (0.13)
Coronary artery disease, n (%)	2 (16.7)	1 (11.1)	2 (22.2)	NS (0.82)
Ischemic cardiomyopathy, n (%)	1 (8.3)	2 (22.2)	1 (11.1)	NS (0.63)
Angina pectoris, n (%)	**3 (25.0)**	**6 (66.7)**	**1 (11.1)**	**< 0.05**
Chronic obstructive pulmonary disease, n (%)	2 (16.7)	0 (0)	2 (22.2)	NS (0.35)
Diabetes mellitus, n (%)	3 (25.0)	1 (11.1)	3 (33.3)	NS (0.52)
Hypertension, n (%)	7 (58.3)	7 (77.8)	8 (88.9)	NS (0.27)
Renal Insufficiency, n (%)	0 (0)	1 (11.1)	1 (11.1)	NS (0.49)
Metabolic disease, n (%)	1 (8.3)	3 (33.3)	3 (33.3)	NS (0.28)
Heart failure, n (%)	0 (0)	1 (11.1)	3 (33.3)	NS (0.08)
Intervention				
Isolated AVR, n (%)	5 (41.7)	3 (33.3)	2 (22.2)	NS (0.65)
Isolated MVR, n (%)	2 (16.7)	0 (0)	1 (11.1)	NS (0.45)
Combined AVR and CABG, n (%)	5 (41.7)	4 (44.4)	2 (22.2)	NS (0.56)
Combined MVR and CABG, n (%)	0 (0)	2 (22.2)	2 (22.2)	NS (0.11)
Combined AVR and MVR, n (%)	0 (0)	0 (0)	2 (22.2)	NS (0.56)

*The three patient groups correspond to control patients (Ctrl), patients with atrial dilatation (AD), and patients with atrial fibrillation (AF). Data are numbers (*n*) and percentages (%), mean ± standard deviation or median [interquartile range], as pertinent. Statistical differences were consistently assessed by Pearson’s Chi Square, ANOVA or Kruskal–Wallis tests. Significant differences are highlighted in bold character. AVR, aortic valve replacement; CABG, coronary artery bypass grafting; LA, left atrium; LVEF, left ventricular ejection fraction; MVR, mitral valve replacement; NS, non-significant.*

Patients were classified into three groups based on AD grades and arrhythmic profile. The control group included patients with no history of AF and normal atrial dimension to mild dilatation (grades 0–1), the AD group patients with no history of AF and moderate to severe dilatation (grades 2–3), and the AF group included patients with documented persistent AF and moderate to severe dilatation.

The investigation was approved by the Ethical Committee for Clinical Experimentation of the Provincial Agency for Health Services of the Autonomous Province of Trento, and conformed to the principles outlined in the declaration of Helsinki. All patients gave written informed consent.

## Materials and Methods

### Sample Processing and RNA Isolation

Small right atrial appendage biopsies (∼30–50 mg) were flash-frozen in pre-chilled liquid isopentane and stored at −80°C until RNA isolation. Each frozen tissue sample was placed in a sterile 15 ml polypropylene tube containing 1.5 ml of pre-cooled Qiazol reagent (Qiagen, Milan, Italy), and subsequently homogenized in ice by a Polytron (Omni-TH International, Kennesaw, GA, United States) at half speed. Total RNA was extracted using miRNeasy mini kit (Qiagen, Milan, Italy) according to the manufacturer’s protocol. RNA concentration and purity were assessed spectrophotometrically by Nanodrop ND-1000 (Thermo Fisher Scientific, Wilmington, DE, United States). RNA integrity was evaluated for each sample using Agilent 2100 Bioanalyzer (Agilent Technologies, Santa Clara, CA, United States) with RNA 6000 Nano Kit. We set RNA Integrity Number threshold for good RNA >5, considering the high stability of miRs (Jung et al., 2010). The extracted amount of total RNA varied among samples, with a median concentration of 125.6 ng/μl (IQR: 103.3–179.2 ng/μl). Integrity was good in all samples with a median value of 7.6 (IQR: 6.8–8.2). RNA aliquots were stored at −80°C until use.

### Reverse Transcription

The total RNA isolated from each tissue sample was reverse-transcribed using miRcury LNA^TM^ Universal cDNA Synthesis kit II (Exiqon, Vedbaek, Denmark), containing 2 μl of Reaction Buffer 5X, 1 μl of Enzyme mix, 10 ng of RNA in a reaction volume of 10 μl, according to the manufacturer’s protocol (Exiqon: Cat. No. 203301, Version 6.1, 04/2015). The reaction conditions were: incubation at 42°C for 60 min, heat-inactivation of the reverse transcriptase at 95°C for 5 min, cooling and storage at 4°C. The retrotranscription was realized by adding a poly-A tail to the mature miR template and synthesizing the cDNA by a poly-T primer with a 3′ degenerate anchor and a 5′ universal tag.

### qPCR

Quantitative polymerase chain reaction (qPCR) assays based on SYBR Green I were performed according to the manufacturer’s protocol (Exiqon: Cat. No. 203403, Version 6.1, 04/2015) on twelve miRs (Supplementary Table S1; hsa-miR-1-3p, hsa-miR-133a-3p, hsa-miR-133b, hsa-miR-21-5p, hsa-miR-29a-3p, hsa-miR-29b-3p, hsa-miR-208a-3p, hsa-miR-208b-3p, hsa-miR-30c-5p, hsa-miR-328-3p, hsa-miR-499a-5p, and hsa-miR-590-5p), which were selected based on their recognized role in cardiovascular disease and AF pathophysiology (Santulli et al., 2014; Luo et al., 2015; Liu et al., 2017; Molina and Voigt, 2017; van den Berg et al., 2017; Wojciechowska et al., 2017). Both forward and reverse primers were miR-specific and optimized with LNA^TM^. qPCR reactions were performed using ExiLENT SYBR^®^ Green master mix (Exiqon, Vedbaek, Denmark) in a CFX384 Real-Time PCR Detection System (Bio-Rad Laboratories, Milan, Italy). The 10 μl PCR reaction contained 4 μl of the diluted cDNA template, 5 μl of SYBR^®^ Green master mix and 1 μl of PCR primer mix. The reaction protocol was as follows: 95°C for 10 min, followed by 40 amplification cycles at 95°C for 10 s and 60°C for 1 min. Each sample was assessed in technical triplicates. Replicates at the qPCR step were used to ensure against failed reactions, so that data points were not missed. qPCR no-template controls were run to set the background level. No sign of contamination was observed. The qPCR quantification cycle (Cq) was determined using Bio-Rad CFX Manager (Bio-Rad Laboratories Inc., Hercules, CA, United States) and single threshold method. Cq values were estimated for each patient and gene as the average of technical triplicates, after outliers’ removal (Cqs that differed more than 1 from the triplicate median). The normalized expression of each miR was calculated by the 2^–ΔΔCq^ method, using as control in each plate the average value of the miR in the control group, and as reference gene the small nucleolar RNA SNORD48, whose stability in human atrial tissue was previously proven (Masè et al., 2017). The fold-change in miR expression in AD/AF groups with respect to the control group was quantified as the ratio of the median values of the normalized expression of AD/AF groups to the control group. MiR expression data in each patient are reported in Supplementary Data Sheet S1.

### Statistical Analysis

Categorical variables were expressed as numbers or percentages, and statistical differences between categorical data were evaluated by Pearson’s Chi Square test. Continuous variables were given as mean ± standard deviation (SD), mean [standard error (SE)], or median (IQR), depending on data normality assessed by Shapiro–Wilk test. Consistently, statistical differences among continuous variables in the three groups were evaluated by one-way Analysis of Variance (ANOVA) or Kruskal–Wallis test, followed by *post hoc* multiple comparison tests with Bonferroni correction among the three patient groups. Given the exploratory nature of the study, no correction for multiple comparisons for the set of analyzed miRs was performed, and each miR was evaluated as an independent hypothesis (Rothman, 1990; Althouse, 2016). Correlation between echocardiographic measures was evaluated by Pearson correlation coefficient (r).

To investigate the relationship between miR expression level, AD, and AF a multivariate approach was used. Specifically, a generalized linear model was fitted to the data, where miR expression was the dependent variable and AD grade and AF presence were the independent variables. Details on multivariate analysis are reported in Supplementary Methods and Supplementary Table S4. All analyses were performed using MATLAB R2017a (The MathWorks, Inc., Natick, MA, United States).

## Results

### Characteristics of Control, AD, and AF Groups

The characteristics of control, AD, and AF groups are reported in Table 1. The control group comprised 12 patients (two females), the AD group nine patients (one female), and the AF group nine patients (three females). Echocardiographic data showed the presence of a statistically significant (*p* < 0.0001) difference in left AD grade, which was significantly higher in AD and AF groups with respect to the control group. Conversely, there was no significant difference in left ventricular ejection fraction values among the groups. The three groups did not present significant differences among demographic or clinical variables, except for the presence of angina pectoris, which was more common in the AD group.

### MicroRNA Expression Profiles in Atrial Dilatation and AF

**FIGURE 1:**
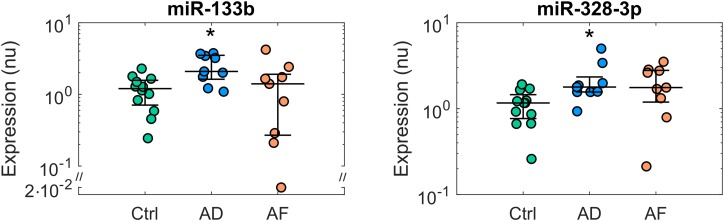
Expression profiles of microRNAs displaying deregulation in patients with atrial dilatation (AD). Comparison of microRNA normalized expression in the control (Ctrl, green dots, *n* = 12), AD (blue dots, *n* = 9), and AF (red dots, *n* = 9) groups. Expression data are shown in logarithmic scale. For each group, dots represent individual expression values, while solid line whiskers represent median and interquartile range. nu, normalized units. ^∗^*p* < 0.05 versus Ctrl group.

**FIGURE 2:**
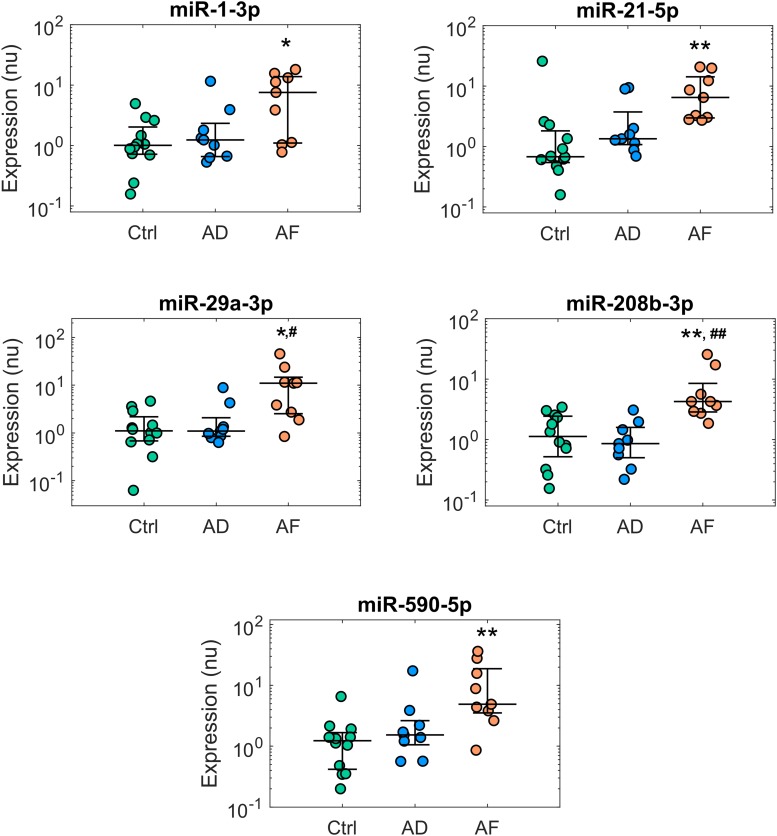
Expression profiles of microRNAs displaying deregulation in patients with atrial fibrillation (AF). Comparison of microRNA normalized expression in the control (Ctrl, green dots, *n* = 12), atrial dilatation (AD, blue dots, *n* = 9), and AF (red dots, *n* = 9) groups. Expression data are shown in logarithmic scale. For each group, dots represent individual expression values, while solid line whiskers represent median and interquartile range. nu, normalized units. ^∗^*p* < 0.05 versus Ctrl group, ^∗∗^*p* < 0.01 versus Ctrl group, ^#^*p* < 0.05 versus AD group, ^##^*p* < 0.01 versus AD group.

The expression profiles of the analyzed miRs in the control, AD, and AF groups of patients are summarized in Supplementary Table S5, while miRs with significant differences among groups (*p* < 0.05) are reported in Figures 1, 2. The comparison of miR expression levels in the three patients’ groups pointed out specific alterations associated with AD and AF. The presence of AD *per se* was associated with significant up-regulation of miR-133b and miR-328-3p with respect to the control group with a fold-change of 1.74 and 1.53, respectively (Figure 1). In the case of miR-328-3p, similar up-regulation was observed in AF patients versus controls, although the difference did not reach statistical significance (fold-change of 1.52, *p* = 0.12 versus control). Differently, in the case of miR-133b, expression levels in AF patients were very similar to those in controls (fold-change of 1.17).

The presence of AF was associated with additional alterations of miR expression (Figure 2). Five miRs, i.e., miR-1-3p, miR-21-5p, miR-29a-3p, miR-208b-3p, and miR-590-5p, showed significant up-regulation in the AF versus control group, with a fold-change ranging from 3.77 for miR-208b-3p to 9.88 for miR-29a-3p. In this group of miRs, the presence of the sole AD was associated only with limited, non-significant effects.

### Relationship Between miR Expression and AD Grade

**FIGURE 3:**
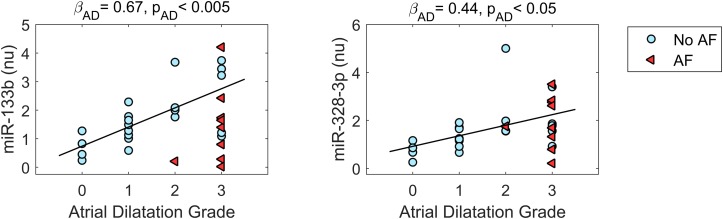
Relationship between microRNA expression and atrial dilatation (AD) grade for microRNAs displaying deregulation in AD. Expression values are plotted as a function of AD grade for patients without AF (no AF, cyan dots, *n* = 21) and patients with AF (AF, red triangles, *n* = 9). The black line represents the regression line (with slope β*_*AD*_* and statistical significance *p*_*AD*_) of microRNA expression on AD grade from multivariate analysis, which pointed out a statistically significant positive association between microRNA expression and AD grade. nu, normalized units.

The relationship between miR expression levels, AD grade, and AF was further investigated by a multivariate approach. In Figure 3, miR expression data are displayed as a function of AD grade for the miRs that displayed significant deregulation in AD, while the results of model fitting are summarized in Supplementary Table S6.

As it can be appreciated in Figure 3, miR-133b and miR-328-3p displayed progressively increasing expression values at larger AD grades. Multivariate analysis confirmed the association between these miRs and AD, where AD grade was a significant (*p*_AD_ < 0.005 and < 0.05, respectively) predictor of miR expression. As shown by the regression lines, an increase of each AD grade led to an increase of 0.67 (0.19) in the normalized expression of miR-133b and of 0.44 (0.20) for miR-328-3p. Nonetheless, the two miRs displayed different relationships with AF. No significant association between miR-328-3p expression and AF presence was observed (*p*_AF_ = 0.60), with an overall model performance of *R*^2^ = 0.18 (*p*_m_ = 0.07). This can be explained by the fact that AD patients and AF patients displayed similar levels of miR-328-3p and similar AD grades (Figures 1, 3), so that alterations in miR-328-3p expression in AF patients could be mainly attributed to the presence of AD. Differently, in the case of miR-133b a significant (*p*_AF_ < 0.05) negative association between miR expression and AF presence was identified, where the latter induced a decrease of normalized expression of −1.25 (0.47). The similar expression values of miR-133b in controls and AF patients (Figures 1, 3) could thus be associated with the concurring effects of AD and AF in the AF group, which resulted in a null global effect. Overall, the model reproduced miR-133b expression variability with an *R*^2^ = 0.31 (*p*_m_ < 0.01).

A different scenario was observed for the miRs displaying altered expression in AF. Multivariate analysis showed AD grade to be a poor predictor of miR expression, which was instead consistently associated with AF presence (Supplementary Table S6).

## Discussion

To our knowledge this is the first study designed to characterize miR expression profiles associated with AD in human atrial tissue. Our results showed that AD *per se* was associated with up-regulation of miR-328-3p and miR-133b, although dilatation produced different expression patterns of these miRs when combined with AF.

### Quantification of miR Expression in Human Atrial Dilatation

In this study, we performed a specific assessment of miR alterations associated with AD in humans by combining RT-qPCR expression profiling and multivariate analysis in atrial tissue samples from cardiac surgery patients with different AD profiles.

Our analysis was focused on a panel of twelve miRs, which were previously indicated for their recognized role in cardiovascular disease (Santulli et al., 2014; Luo et al., 2015; van den Berg et al., 2017), and thus that could be of major interest in the setting of AF. Despite the small number of miRs analyzed, we were able to point out the up-regulation of two miRs in the AD group. These results confirmed our initial hypothesis that AD *per se* was associated with an altered miR profile, but prompt, as well, future studies with high throughput approaches to identify further alterations.

As concerns the target population, while a significant body of studies investigated miR alterations in AF patients (Santulli et al., 2014; Luo et al., 2015; van den Berg et al., 2017), our study was focused on the sinus rhythm, which was subdivided based on AD grades to extract the specific miR profile associated with dilatation. The evaluation of atrial enlargement was performed on the left atrium to avail from standardized and reliable criteria for dilatation assessment, and was expressed in terms of AD grades to reduce potential uncertainty associated with echocardiographic assessment (Lang et al., 2015). The presence of left AD may, however, be indicative of similar, albeit reduced, alterations in the right atrium, as suggested by the correlation between left and right atrial volumes observed in the subset of patients where right atrial measures were available. Of note, the control group in our study included patients with slight AD. This is in agreement with recent imaging studies (Petersen et al., 2017) showing that the normal reference range that allows to differentiate between normal and pathological states is larger than those suggested in clinical guidelines (Lang et al., 2006). The comparison of miR expression levels in controls versus AD patients highlighted an altered miR profile in the AD group, bringing awareness on the heterogeneity of the sinus rhythm group generally used as comparator in AF expression studies. As suggested by our results, the presence of patients with different AD grades in the sinus rhythm group may increase the variability of miR expression values and constitute a potential inconsistency factor among miR expression studies.

To further investigate the association between miR expression, AD and AF, a multivariate analysis of miR expression as a function of AD grade and AF presence was applied. Although few previous studies analyzed miR correlation with AD (Liu et al., 2014a; Soeki et al., 2016), our approach aimed to distinguish a direct association with AD with respect to AF-mediated associations. Of note, the analysis enabled us to suggest opposite associations of AD and AF with miR-133b expression, and the lack of a direct involvement of AD in miR-1-3p, miR-29a-3p, miR-208b-3p, and miR-590-5p deregulation.

### MicroRNA Alterations in Atrial Dilatation and AF: Implications for Atrial Remodeling

Expression data comparison corroborated by multivariate analysis results showed that the expression of miR-328-3p was mainly associated with AD grade, so that similar expression values were observed for AD and AF patients, who displayed similar dilatation grades. Differently, miR-133b expression levels were associated with both AD grade and AF presence, the two factors exerting opposite modulation effects, resulting in a null overall effect in AF patients. Although the deregulations of these miRs in AD were of small entity (less than 2-fold), they may still underlie a biologically relevant mechanism, given the small variability of these miRs and the fact that a single miRNA may alter an entire biological pathway by inhibiting numerous mRNAs at once through partial base complementarity. Up-regulation of miR-328-3p in AD is consistent with previous findings in a mouse model of cardiac hypertrophy (Li C. et al., 2014), in a canine AF model (Lu et al., 2010), and in human atrial samples from AF patients with rheumatic heart disease (Lu et al., 2010). As well, local left atrial appendage plasma levels of miR-328 were shown to be higher in AF patients than controls, and to display positive correlation with left atrial volume index and extension of low voltage areas (Soeki et al., 2016). It should be noticed that in our study the up-regulation pattern of miR-328-3p in AF patients did not reach statistical significance, which may be partially related to the small number of patients analyzed and the higher variability of miR expression in the AF group.

Considering miR-133b, its up-regulation in AD patients is in agreement with experimental studies in canine, rabbit, and ovine models of heart failure (Belevych et al., 2011; Olivito et al., 2014; Wong et al., 2017), a condition associated with atrial stretch and increased atrial pressure. As well the negative association of miR-133b with the presence of AF is consistent with previous studies, showing miR-133 down-regulation in canine AF (Shan et al., 2009; Li et al., 2012), and in atrial samples from AF patients with smoking versus non-smoking habits (Shan et al., 2009).

As concerns the regulatory role of the two miRs, experimental studies suggested the involvement of miR-328 in calcium homeostasis (Lu et al., 2010; Li C. et al., 2014), where its forced expression led to diminished L-type calcium current (I_Ca,L_), shortening of atrial action potential duration, and increased AF vulnerability (Lu et al., 2010). As well, miR-133 family was shown to act as regulator of calcium and potassium currents (Belevych et al., 2011; Shan et al., 2013), with either cardio-protective (Liu et al., 2017) and pro-arrhythmic (Belevych et al., 2011) effects reported. Interestingly, altered calcium homeostasis and, in particular, diminished I_Ca,L_ density is a recognized hallmark and causal factor of the electrical remodeling induced by AF, but also by the sole AD. In the three positive feedback-loops of atrial remodeling proposed by Allessie et al. (2002), reduction of I_Ca,L_ was considered the primary cause of electrical remodeling, leading to shortening of the refractory period, and of contractile remodeling, which in turn stimulated structural remodeling. Similarly, reduced I_Ca,L_ was a common fingerprint in experimental models of dilatation (Li et al., 1999; Deroubaix et al., 2004) and in cardiac myocytes from dilated hearts (Le Grand et al., 1994; Dinanian et al., 2008), although variable effects on the refractory period were observed in different experimental models (Li et al., 1999; Deroubaix et al., 2004). We can thus speculate that the observed up-regulation of miR-328-3p and miR-133b in AD patients may support the role of a post-transcriptional mechanism in the reduction of L-type calcium currents in dilatation-induced electrical remodeling (Van Wagoner, 2008). On the other hand, since up-regulation of miR-133b was previously shown to reduce the expression of the delayed rectifier and inward rectifier potassium channel subunits, leading to refractory period prolongation (Shan et al., 2013), the differential regulation of miR-133b by AD and AF may potentially contribute to the different modulation of the refractory period observed in the two remodeling types.

In addition to the alterations observed in AD, our analysis pointed out further miR alterations related to AF, which may be involved in the formation of a proarrhythmic substrate. Similarly to our results, up-regulation of miR-21-5p, miR-29a-3p, and miR-208b in AF patients was observed in previous experimental and clinical studies (Adam et al., 2012; Barana et al., 2014; Torrado et al., 2015; Cañón et al., 2016; Zhao et al., 2016), which suggested a role of these miR in the AF-mediated reduction of I_Ca,L_. In addition, up-regulation of miR-1-3p in AF was observed in experimental tachy-pacing AF models (Jia et al., 2013; Torrado et al., 2015), where it contributed to AF-induced electrical remodeling by targeting KCNE1 and KCNB2 genes (Jia et al., 2013). Altered expression of miR-21-5p, miR-29a-3p, and miR-590-5p may contribute to structural remodeling, modulating, albeit with different effects, collagen production and fibrosis (Shan et al., 2009; Adam et al., 2012; Santulli et al., 2014; Luo et al., 2015; van den Berg et al., 2017). It should be noticed that alterations of miR-1-3p, miR-29a-3p, and miR-590-5p in human AF were not consistently reported in previous studies. MiR-29a was found down-regulated in right atrial samples from AF patients by microarray analysis, albeit the results were not validated by RT-qPCR precluding a detailed comparison (Liu et al., 2014b). Either down-regulation (Girmatsion et al., 2009; Li J. et al., 2014; Santulli et al., 2014) and unaltered expression (Lu et al., 2010; Cooley et al., 2012) of miR-1 were observed in human AF. Down-regulation of miR-590 was observed in atrial samples from AF patients with smoking versus non-smoking habits (Shan et al., 2009). Differences in miR profiles may arise from different biological and technical designs among studies, from the choice of the reference genes (Masè et al., 2017), and from heterogeneity among the studied AF populations.

### Study Limitations and Future Perspectives

This observational study has several limitations. The main limitations are the small sample size and the focus on twelve miRs. Further significant differences in miR expression may have been observed in larger populations and by high throughput approaches. The studied population presented inhomogeneities in comorbidities, e.g., angina pectoris and mitral valve disease, which may have acted as confounding factors, contributing to the variability of miR expression. In addition, our population did not include AF patients with normal atrial dimensions. Due to the restrictions imposed by the cardiac surgery setting, only right atrial appendage specimens were analyzed, although differences in miR expression may be present among atrial regions. The study was limited to miR expression profiling by RT-PCR, while no assessment of potential target gene expression, nor miR manipulation to induce target gene alterations was performed. Suggested mechanistic implications and causal link of the observed miR alteriations need to be tested in future studies. Our results may also stimulate future investigations on biomarkers, which should test whether miR alterations in atrial tissue are also detectable in circulating miRs, as well as assess miR discrimination power by advanced techniques, such as principal component and cluster analyses.

## Conclusion

In this study, we analyzed for the first time the specific miR expression profiles associated with human AD. Our results showed that chronic atrial stretch *per se* was related to up-regulation of miR-328-3p and miR-133b, albeit the two miRs presented different relationship with AF. These results may pave the way to future experimental studies to test the involvement of post-transcriptional mechanisms in the stretch-induced formation of a pro-arrhythmic substrate.

## Data Availability

All datasets generated for this study are included in the manuscript and/or the Supplementary Files.

## Ethics Statement

The studies involving human participants were reviewed and approved by the Ethical Committee for Clinical Experimentation of the Provincial Agency for Health Services of the Autonomous Province of Trento. The patients and participants provided their written informed consent to participate in this study.

## Author Contributions

MM, MD, and FR conceived and designed the study. MG, LA, ED’A, MNG, and AG contributed to the data acquisition. MM and FT performed the data analysis. MM, AG, MD, and FR contributed to the data interpretation. MM prepared the figures and drafted the manuscript. MD and FR edited and revised the manuscript. All authors approved the final version of the manuscript.

## Conflict of Interest Statement

The authors declare that the research was conducted in the absence of any commercial or financial relationships that could be construed as a potential conflict of interest.
