# Supplementary material for: Upregulation of miR-133b and miR-328 in Patients With Atrial Dilatation: Implications for Stretch-Induced Atrial Fibrillation
Source: Front Physiol. 2019 Sep 10;10:1133. doi: 10.3389/fphys.2019.01133 (PMC6748158; doi:10.3389/fphys.2019.01133)
Supplement: Supplementary file 1 [file Presentation_1.zip › Supplementary Material/Supplementary Tables Figures Methods.pdf]

Supplementary Table S1. Overview of the analyzed microRNAs. List of oligonucleotide names and target sequences (miRCURY LNA™ PCR primers set). All sequences are presented in a 5′-3′ orientation.

Oligonucleotide name	Target sequence (5’-3’)
**hsa-miR-1-3p**	UGGAAUGUAAAGAAGUAUGUAU
**hsa-miR-133a-3p**	UUUGGUCCCCUUCAACCAGCUG
**hsa-miR-133b**	UUUGGUCCCCUUCAACCAGCUA
**hsa-miR-21-5p**	UAGCUUAUCAGACUGAUGUUGA
**hsa-miR-29a-3p**	UAGCACCAUCUGAAAUCGGUUA
**hsa-miR29b-3p**	UAGCACCAUUUGAAAUCAGUGUU
**hsa-miR-208a-3p**	AUAAGACGAGCAAAAAGCUUGU
**hsa-miR-208b-3p**	AUAAGACGAACAAAAGGUUUGU
**hsa-miR-30c-5p**	UGUAAACAUCCUACACUCUCAGC
**hsa-miR-328-3p**	CUGGCCCUCUCUGCCCUUCCGU
**hsa-miR-499a-5p**	UUAAGACUUGCAGUGAUGUUU
**hsa-miR-590-5p**	GAGCUUAUUCAUAAAAGUGCAG
**SNORD48**	AGTGATGATGACCCCAGGTAACTCTGAGTGTG TCGCTGATGCCATCACCGCAGCGCTCTGACC

Supplementary Table S2. Reference limits and partition values for left atrial dimensions/volumes. Adapted from Lang et al. (2006).

	Women				Men			
	Reference Range<br>Grade 0	Mildly Abnormal<br>Grade 1	Moderately Abnormal<br>Grade 2	Severely Abnormal<br>Grade 3	Reference Range<br>Grade 0	Mildly Abnormal<br>Grade 1	Moderately Abnormal<br>Grade 2	Severely Abnormal<br>Grade 3
**Atrial dimensions**								
**AP diameter (cm)**	2.7-3.8	3.9-4.2	4.3-4.6	≥4.7	3.0-4.0	4.1-4.6	4.7-5.2	≥5.2
**LA area (cm^2)**	≤20	20-30	30-40	>40	≤20	20-30	30-40	>40
**LA volume (ml)**	22-52	53-62	63-72	≥73	18-58	59-68	69-78	≥79
**LA volume/BSA (mol/m^2)**	22±6	29-33	34-39	≥40	22±6	29-33	34-39	≥40

AP, anteroposterior; BSA, body surface area; LA, left atrium.

Reference

Lang, R.M., Bierig, M., Devereux, R.B., Flachskampf, F.A., Foster, E., Pellikka, P.A., Picard, M.H., Roman, M.J., Seward, J., Shanewise, J., et al. (2006). Recommendations for chamber quantification. Eur J Echocardiogr 7, 79–108.

Supplementary Table S3. Echocardiographic measures performed in the study population, and assessment of left atrial dilatation grade according to the criteria reported in Table S2.

Patients	Sex	Group	LA dimensions		LA dilatation grade	RA dimensions		LVEF
			AP Diameter, cm (Area, cm^2)	Volume, ml (Volume/BSA, ml/m^2)		Minor axis, cm (Area, cm^2)	Volume, ml (Volume/BSA, ml/m^2)	
**P1**	M	Ctrl	3.7	28	0	-	29	66
**P2**	M	Ctrl	-	62	1	-	-	59
**P3**	M	Ctrl	3.5	-	0	-	-	64
**P4**	M	AD	-	95 (44)	3	-	-	64
**P5**	M	AD	4.8	-	2	5.4	-	60
**P6**	M	AF	-	149 (82)	3	-	-	37
**P7**	M	Ctrl	4.1	-	1	-	-	67
**P8**	M	AF	5.3	(84)	3	-	-	70
**P9**	M	AD	-	79 (45)	3	-	45 (25)	65
**P10**	M	AF	6.2	-	3	-	-	73
**P11**	M	AD	-	123 (63)	3	(21)	64 (33)	78
**P12**	M	Ctrl	(26)	-	1	-	-	59
**P13**	M	Ctrl	-	(31)	1	-	-	61
**P14**	M	AD	-	(37)	2	-	-	55
**P15**	M	Ctrl	-	41 (19)	0	(13)	25 (12)	58
**P16**	M	AD	-	(38)	2	-	-	40
**P17**	M	Ctrl	-	68	1	(18)	56	68
**P18**	M	AD	5.2	101	3	-	48	52
**P19**	M	AF	(31)	-	2	-	-	56
**P20**	M	AF	-	110 (51)	3	-	-	57
**P21**	F	AF	-	73 (44)	3	-	41 (28)	67
**P22**	F	AF	(54)	-	3	(40)	-	59
**P23**	M	Ctrl	4.1	59	1	-	34	71
**P24**	M	AF	5.2	(58)	3	-	(55)	35
**P25**	F	AD	-	71 (39)	2	(16)	37 (21)	60
**P26**	M	Ctrl	4.5 (27)	-	1	4.1	-	70
**P27**	F	AF	(41)	198 (111)	3	(27)	91 (51)	55
**P28**	F	Ctrl	-	62 (33)	1	-	-	57
**P29**	F	Ctrl	-	(21)	0	-	-	66
**P30**	M	AD	-	91	3	-	-	61

AD, atrial dilatation; AP, anteroposterior; AF, atrial fibrillation; BSA, body surface area; Ctrl, control group; F, female; LA, left atrium; LVEF, left ventricular ejection fraction; M, male; RA, right atrium.

Supplementary Methods on Multivariate Analysis

The relationship between miR expression level, atrial dilatation (AD), and atrial fibrillation (AF) was analyzed by multivariate analysis. Specifically, a generalized linear model was fitted to the data, where miR expression was the dependent variable and AD grade and AF presence were the independent variables, according to the expression:

$$\text{miR expression} \sim Int + \beta_{AD} \cdot \text{AD grade} + \beta_{AF} \cdot \text{AF presence} \qquad (1)$$

where *Int* is the intercept of the model, AD grade is a discrete variable that takes values between 0 and 3, AF presence is a logical variable, equal to 0 in absence of AF and to 1 in presence of AF, β_{AD} and β_{AF} are the coefficients (slopes) of the model associated with AD grade and AF variables, respectively. In particular, each slope quantifies the change of the dependent variable associated with a change of one unit in the corresponding independent variable, and was assumed as a measure of the strength of the association between the dependent variable and the independent variable. The statistical significance of each association versus the null hypothesis of no effect (i.e., coefficient equal to zero) was assessed in terms of the p-values of the slopes (p_{AD} and p_{AF}, respectively). Low p-values indicated that the predictor was a meaningful addition to the model, because changes in the predictor's values were related to changes in the response variable. The overall performance of the model in describing the variability of the dependent variable was assessed in terms of the ordinary (unadjusted) R^2 statistics, which quantifies the proportion of variation of the dependent variable explained by the model, and the adjusted R^2 statistics, which is corrected for the number of the coefficients. The statistical significance of the overall model in describing the data versus the null hypothesis of an intercept-only, constant model was assessed in terms of the *F*-statistic and corresponding p-value (p_m). A p-value < 0.05 was considered statistically-significant. All analysis were performed in Matlab R2017a (The MathWorks, Inc., MA), using *'fitglm'* function.

Supplementary Table S4. Description of multivariate analysis outputs.

**Parameter**	**Meaning**
Int	Intercept of the generalized linear model.
$\boldsymbol{\beta_{AD}}$	Coefficient (slope) of the generalized linear model associated with atrial dilatation (AD) grade, quantifying the strength of the association between the predictor (AD grade) and the dependent variable (miR expression).
$\boldsymbol{p_{AD}}$	Statistical significance of $\boldsymbol{\beta_{AD}}$, indicating the significance of AD grade as predictor versus the null-hypothesis of no effect.
$\boldsymbol{\beta_{AF}}$	Coefficient (slope) of the linear model associated with the presence of atrial fibrillation (AF), quantifying the strength of the association between the predictor (AF presence) and the dependent variable (miR expression).
$\boldsymbol{p_{AF}}$	Statistical significance of $\boldsymbol{\beta_{AF}}$, indicating the significance of AF presence as predictor versus the null-hypothesis of no effect.
$\boldsymbol{R^2}$	Ordinary (unadjusted) R-squared or coefficient of multiple determination, quantifying the proportion of variation of the dependent variable explained by the model.
$\boldsymbol{R^2}$***-adjusted***	Adjusted R-square, corrected for the number of coefficients.
$\boldsymbol{F, p_m}$	F-statistics and p-value of the overall model versus the the null-hypothesis of an intercept-only, constant model.

Supplementary Table S5. Comparison of microRNA expression profiles in the control (Ctrl), atrial dilatation (AD), and atrial fibrillation (AF) groups. Expression data are median [interquartile range], and are given in normalized units (nu). Fold-changes in AD and in AF groups are calculated as the ratio of their median normalized expression values to the median normalized expression of the Ctrl group. For each miR, statistical differences among the three groups were assessed by Kruskall-Wallis test, followed by post-hoc multiple comparison tests with Bonferroni correction. All tests were performed in Matlab R2017a (The MathWorks, Inc., MA) using *'kruskalwallis'* and *'multcompare'* functions. Significant differences are highlighted in bold character. NS, non-significant.

**microRNAs**	**Ctrl Group (n=12)**	**AD Group (n=9)**		**AF group (n=9)**		
	**Expression (nu)**	**Expression (nu)**	**Fold-change**	**Expression (nu)**	**Fold-change**	**p-value**
**miR-1-3p**	1.00 [0.72-2.03]	1.24 [0.66-2.33]	1.24	**7.57 [1.10-13.76]***	**7.57**	<0.05
**miR-133a-3p**	0.71 [0.56-1.63]	1.33 [0.97-1.93]	1.87	2.56 [1.16-4.24]	3.61	NS (0.13)
**miR-133b**	1.20 [0.71-1.57]	**2.09 [1.62-3.50]***	**1.74**	1.40 [0.27-1.91]	1.17	<0.05
**miR-21-5p**	0.68 [0.54-1.82]	1.34 [1.07-3.73]	1.97	**6.48 [2.97-14.15]****	**9.53**	<0.005
**miR-29a-3p**	1.11 [0.68-2.17]	1.10 [0.85-2.08]	0.99	**10.97 [2.52-14.63]***,#	**9.88**	<0.05
**miR-29b-3p**	1.21 [0.39-1.61]	1.05 [0.84-1.88]	0.87	2.55 [1.44-5.08]	2.11	NS (0.08)
**miR-208a-3p**	1.46 [0.41-3.12]	2.04 [1.07-3.29]	1.40	1.97 [0.39-4.72]	1.35	NS (0.84)
**miR-208b-3p**	1.13 [0.52-2.43]	0.86 [0.50-1.58]	0.76	**4.26 [2.87-8.52]****,##	**3.77**	<0.005
**miR-30c-5p**	1.14 [0.63-1.65]	1.47 [1.06-1.68]	1.29	1.64 [1.16-4.51]	1.44	NS (0.16)
**miR-328-3p**	1.16 [0.76-1.45]	**1.78 [1.56-2.33]***	**1.53**	1.76 [1.19-2.78]	1.52	<0.05
**miR-499a-5p**	1.01 [0.47-2.28]	1.75 [0.57-2.28]	1.73	2.56 [0.86-4.06]	2.53	NS (0.35)
**miR-590-5p**	1.23 [0.42-1.67]	1.53 [1.05-2.63]	1.24	**4.90 [3.52-18.85]****	**3.98**	<0.01

*, p<0.05 versus Ctrl, **, p<0.01 versus Ctrl, #, p<0.05 versus AD, ##, p<0.01 versus AD.

Supplementary Table S6. Relationship between miR expression, atrial dilatation (AD) and atrial fibrillation (AF). miR expression data were fitted by a generalized linear model, assuming AD grade and AF presence as independent variables. For each model the intercept (Int) and slopes (β_{AD}, β_{AF}) of the independent variables are reported with the corresponding standard error (SE) and statistical significance (p, p_{AD}, p_{AF}). Overall model performance is characterized in terms of R^2, R^2-adjusted, F-stastistics and statistical significance (p_m). Significant associations with AD grade and/or AF presence are highlighted in bold character.

**microRNAs**	**Int (SE)**<br>**p-value**	**β_{AD} (SE)**<br>**p-value**	**β_{AF} (SE)**<br>**p-value**	**R^2**	**R^2-adjusted**	**F-statistics vs constant model**	**p_m-value**
**miR-1-3p**	1.37 (1.58)<br>p = 0.39	0.37 (0.86)<br>p_{AD} = 0.67	**5.59 (2.08)**<br>**p_{AF} < 0.05**	0.33	0.28	6.66	<0.005
**miR-133a-3p**	2.92 (2.00)<br>p = 0.16	-0.21 (1.10)<br>p_{AD} = 0.85	2.05 (2.65)<br>p_{AF} = 0.45	0.03	-0.05	0.35	0.71
**miR-133b**	0.74 (0.36)<br>p <0.05	**0.67 (0.19)**<br>**p_{AD} < 0.005**	**-1.25 (0.47)**<br>**p_{AF} < 0.05**	0.31	0.26	6.18	<0.01
**miR-21-5p**	2.26 (2.35)<br>p = 0.34	0.53 (1.29)<br>p_{AD} = 0.69	5.06 (3.10)<br>p_{AF} = 0.11	0.17	0.11	2.74	0.08
**miR-29a-3p**	2.23 (2.95)<br>p = 0.46	-0.25 (1.62)<br>p_{AD} = 0.88	**10.98 (3.90)**<br>**p_{AF} < 0.01**	0.29	0.24	5.64	<0.01
**miR-29b-3p**	1.87 (0.86)<br>p < 0.05	-0.11 (0.47)<br>p_{AD} = 0.82	2.06 (1.14)<br>p_{AF} = 0.08	0.14	0.07	2.16	0.14
**miR-208a-3p**	1.26 (2.87)<br>p = 0.66	0.60 (1.57)<br>p_{AD} = 0.70	3.65 (3.80)<br>p_{AF} = 0.35	0.08	0.01	1.14	0.34
**miR-208b-3p**	1.17 (1.69)<br>p = 0.50	0.11 (0.92)<br>p_{AD} = 0.91	**6.09 (2.23)**<br>**p_{AF} < 0.05**	0.31	0.25	5.96	<0.01
**miR-30c-5p**	1.13 (0.58)<br>p = 0.06	0.22 (0.32)<br>p_{AD} = 0.49	1.17 (0.76)<br>p_{AF} = 0.14	0.19	0.13	3.12	0.06
**miR-328-3p**	0.92 (0.36)<br>p < 0.05	**0.44 (0.20)**<br>**p_{AD} < 0.05**	-0.25 (0.48)<br>p_{AF} = 0.60	0.18	0.12	2.97	0.07
**miR-499a-5p**	1.05 (0.80)<br>p = 0.20	0.46 (0.44)<br>p_{AD} = 0.30	0.77 (1.05)<br>p_{AF} = 0.47	0.12	0.06	1.92	0.17
**miR-590-5p**	1.19 (2.79)<br>p = 0.67	0.77 (1.53)<br>p_{AD} = 0.62	**8.32 (3.69)**<br>**p_{AF} < 0.05**	0.27	0.22	5.07	<0.05

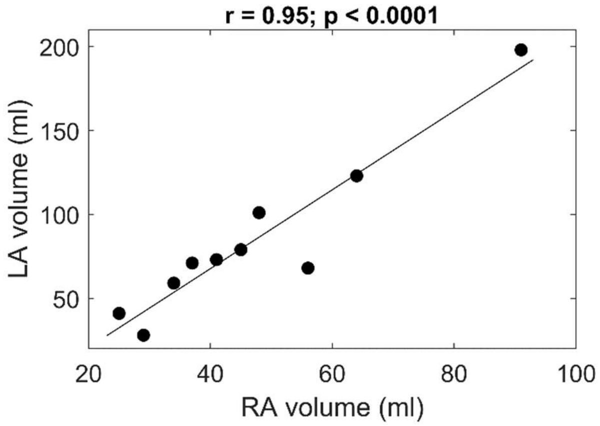

Supplementary Figure S1. Association between echocardiographic measures. A strong, positive, statistically-significant correlation was observed between left and right atrial volumes. The black line represents the linear regression fit, given by $y=-26.22+2.35 \cdot x$ ($n=10$). r: Pearson correlation coefficient; p: p-value.
